# Assessment of the gorilla gut virome in association with natural simian immunodeficiency virus infection

**DOI:** 10.1186/s12977-018-0402-9

**Published:** 2018-02-05

**Authors:** Mirela D’arc, Carolina Furtado, Juliana D. Siqueira, Héctor N. Seuánez, Ahidjo Ayouba, Martine Peeters, Marcelo A. Soares

**Affiliations:** 1 Instituto Nacional de Câncer (INCA), Rio de Janeiro, Brazil; 2 Universidade Federal do Rio de Janeiro (UFRJ), Rio de Janeiro, Brazil; 3 UMI233/INSERM1175 Institut de Recherche pour le Développement (IRD), University of Montpellier, Montpellier, France

**Keywords:** Gorilla, SIVgor, Pathogenesis, Virome, Dysbiosis

## Abstract

**Background:**

Simian immunodeficiency viruses (SIVs) of chimpanzees and gorillas from Central Africa crossed the species barrier at least four times giving rise to human immunodeficiency virus type 1 (HIV-1) groups M, N, O and P. The paradigm of non-pathogenic lentiviral infections has been challenged by observations of naturally infected chimpanzees with SIVcpz associated with a negative impact on their life span and reproduction, CD4^+^ T-lymphocyte loss and lymphoid tissue destruction. With the advent and dissemination of new generation sequencing technologies, novel promising markers of immune deficiency have been explored in human and nonhuman primate species, showing changes in the microbiome (dysbiosis) that might be associated with pathogenic conditions. The aim of the present study was to identify and compare enteric viromes of SIVgor-infected and uninfected gorillas using noninvasive sampling and ultradeep sequencing, and to assess the association of virome composition with potential SIVgor pathogenesis in their natural hosts.

**Results:**

We analyzed both RNA and DNA virus libraries of 23 fecal samples from 11 SIVgor-infected (two samples from one animal) and 11 uninfected western lowland gorillas from Campo-Ma’an National Park (CP), in southwestern Cameroon. Three bacteriophage families (Siphoviridae, Myoviridae and Podoviridae) represented 67.5 and 68% of the total annotated reads in SIVgor-infected and uninfected individuals, respectively. Conversely, mammalian viral families, such as Herpesviridae and Reoviridae, previously associated with gut- and several mammalian diseases were significantly more abundant (p < 0.003) in the SIVgor-infected group. In the present study, we analyzed, for the first time, the enteric virome of gorillas and their association with SIVgor status. This also provided the first evidence of association of specific mammalian viral families and SIVgor in a putative dysbiosis context.

**Conclusions:**

Our results suggested that viromes might be potentially used as markers of lentiviral disease progression in wild gorilla populations. The diverse mammalian viral families, herein described in SIVgor-infected gorillas, may play a pivotal role in a disease progression still unclear in these animals but already well characterized in pathogenic lentiviral infections in other organisms. Larger sample sets should be further explored to reduce intrinsic sampling variation.

## Background

The human immunodeficiency virus (HIV) types 1 and 2 have arisen from multiple zoonotic transmissions of simian immunodeficiency viruses (SIVs) circulating in African non-human primates (NHP) to humans. SIVs from chimpanzees and gorillas from Central Africa crossed the species barrier at least four times giving rise to HIV-1 groups M, N, O, and P [1, 2]. Likewise, at least nine independent transmissions between sooty mangabeys from West Africa and humans have already been described, originating the different HIV-2 groups found in humans [3–5].

Although SIVs have been referred to as immunodeficiency viruses, the clinical manifestations in SIV-infected hosts were not reported during the first decades when natural infections were initially described in the wild and in naturally infected animals, captive sooty mangabeys and African green monkeys kept in zoos or primate centers. SIV lineages have already been documented in more than 40 species of African NHP [6]. Despite high SIV viral loads, individuals of these species have been initially reported as effective controllers of disease progression [7–9]. However, following the experimental or accidental SIV infections of Asian macaques (that are not natural reservoirs of lentiviruses), these latter primates developed an AIDS-like disease that was very similar to the human condition [10, 11].

The paradigm of non-pathogenic lentiviral infections has been recently challenged by observations of wild chimpanzees (*P. t. schweinfurthii*) in Gombe, Tanzania, in which infection by their natural SIV strain (SIVcpz*Pts*) was associated with a negative impact on their life span and reproduction [12, 13]. Moreover, retrospective analysis of conserved tissues from dead SIVcpz*Pts*-infected animals showed signs of immune deficiency similar to those found in AIDS-affected humans [13]. In addition, SIVcpz infection has also been associated with CD4^+^ T lymphocyte loss and lymphoid tissue destruction (alike HIV infection), leading to premature death in a naturally infected chimpanzee in a sanctuary in Cameroon [14]. Altogether, these findings underscored the need for further studies of wild NHP populations, particularly among apes, because of their highly endangered status and the potential impact of SIV infection on their survival and eventual population decline [1, 7, 15]. However, the study of these wild populations is particularly difficult and can only be carried out by noninvasive sampling.

With the advent and dissemination of next generation sequencing (NGS) technologies, novel and promising markers of immune deficiency have been explored in humans and NHP species, notably changes in the microbiome (dysbiosis) potentially associated with pathogenic conditions. In humans, HIV infection is markedly associated with expansion of enteric adenoviruses and significant loss of enteric bacterial diversity and richness, clearly involved in disease progression [16]. Similar evidence of microbiome instability was found in naturally infected chimpanzees in Gombe [17]. To present, there is no available data on the pathogenicity of SIV in gorillas. Moeller et al. [18] examining 186 fecal samples of wild gorillas from Cameroon, did not find association between SIV status and specific patterns of the gut bacteriome. Nevertheless, as gorillas are infected with SIVgor through cross-species transmission of SIVcpz from chimpanzees, it is plausible that SIVgor might negatively impact the health of gorillas in the wild [2].

By combining noninvasive sampling and NGS approaches, Handley et al. [19] reported the expansion of the enteric virome associated with disease progression in an AIDS-like context in rhesus macaques, but not with the nonpathogenic SIV infection of African green monkeys. These findings showed that pathogenic SIV infection was associated with an enteropathy that contributed to the AIDS-like disease progression seen in macaques [19]. In this report, we used a similar strategy for identifying enteric virome disturbances as dysbiosis markers in wild gorillas to understand the potential impact of SIVgor infection in their natural hosts. We identified and compared enteric viromes of SIVgor-infected and uninfected gorillas using noninvasive sampling and NGS to assess the relationship of their viromes with potential SIVgor pathogenesis. Here we present the first virome description of SIVgor-infected and uninfected gorillas, identifying viral family profiles in each studied group. Furthermore, we herein provide the first evidence of association of specific mammalian virus families (such as Adenoviridae, Herpesviridae and Reoviridae) with presence of SIVgor in a putative dysbiosis context in wild animals.

## Methods

### Sample collection

Twenty-three fecal samples from 22 wild western lowland gorillas (*Gorilla gorilla gorilla*) were studied. Samples were collected between May 2008 and February 2014 in Campo Ma’an National Park (CP), Cameroon, in a long-term follow-up project of gorilla communities in this region, where we previously reported 29% of SIVgor-infected animals in some groups [20, 21]. These samples, from 11 SIVgor-infected and 11 uninfected individuals, had been previously analyzed with serological and/or molecular tools for confirming or excluding SIVgor infection [2, 20–22] (Table 1). Animals were individualized with microsatellite analysis as previously described [1, 21]. SIVgor viral load (VL) was measured with a previously reported real-time RT-qPCR assay capable of quantifying all HIV-1 groups and a wide diversity of SIVcpz and SIVgor strains [23]. The fecal samples selected for the study also included being the most fresh (the shortest elapsed time from deposition to collection), having a good amount of material and also covered a wide range of VL (including the highest VL ever measured in an infected gorilla). VL estimates of SIVgor ranged from 20 to 31,497 copies per mL (cp/mL) (Table 1). Two sequential samples were collected within a time interval of approximately 1 year from a single gorilla (CPg-ID074), one sample (CP8789) in 2012, with an estimated VL of 31,497 cp/mL, and another (CP9725) in 2013, with a VL of 4924 cp/mL.

**Table 1:** Description of noninvasive samples used in this study

Lab code	ID^a^	Collection date (dd/mm/yyyy)	Estimated freshness (h)	SIV-specific RT-PCR^b^	SIVgor VL (cp/mL)^c^	SIV serology	GPS coordinates
CP3376	CPg-ID046	12/05/2008	11	NA	NA	NEG	2.334617°N 10.191633°W
CP3385	CPg-ID059	12/05/2008	11	NA	NA	NEG	2.334617°N 10.191633°W
CP3403	CPg-ID030	12/05/2008	ND	gp41/polmini	45	POS	2.334444°N 10.183889°W
CP3409	CPg-ID031	12/05/2008	10	NEG	20	POS	2.33055°N 10.201833°W
CP3453	CPg-ID090	12/10/2008	22	NA	NA	NEG	2.34425°N 10.229833°W
CP3469	CPg-ID034	12/10/2008	ND	gp41	4.217	POS	2.341389°N 10.211389°W
CP5781	CPg-ID066	27/04/2010	14	gp41	1.095	POS	2.325°N 10.1781°W
CP5819	CPg-ID017	30/04/2010	6	NA	NA	NEG	2.32865°N 10.168633°W
CP6535	CPg-ID142	17/03/2011	8	NA	NEG	NEG	2.34647°N 10.28754°W
CP7880	CPg-ID086	27/01/2012	12	NA	NEG	NEG	2.34329°N 10.18654°W
CP7885	CPg-ID004	27/01/2012	12	gp41	655	POS	2.34329°N 10.18654°W
CP7890	CPg-ID005	27/01/2012	12	gp41	2.346	POS	2.34329°N 10.18654°W
CP8084	CPg-ID006	23/04/2012	24	gp41	1.242	POS	2.32763°N 10.18388°W
CP8789	CPg-ID074^d^	12/11/2012	12	gp41/gp120 (V1–V4)	31.497	POS	2.34434°N 10.25066°W
CP8797	CPg-ID021	12/11/2012	12	NA	NEG	NEG	2.34434°N 10.25066°W
CP8804	CPg-ID114	12/11/2012	8	NA	NEG	NEG	2.34362°N 10.25137°W
CP9667	CPg-ID041	13/07/2013	14	gp41/polmini	2.460	POS	2.32650°N 10.20193°W
CP9679	CPg-ID035	13/07/2013	6	polmini	700	POS	2.32650°N 10.20193°W
CP9683	CPg-ID108	13/07/2013	6	NA	NA	NEG	2.32650°N 10.20193°W
CP9686	CPg-ID102	13/07/2013	6	NA	NA	NEG	2.32650°N 10.20193°W
CP9725	CPg-ID074^d^	27/10/2013	12	gp41/gp120/polmini	4.924	POS	2.31423°N 10.19226°W
CP9852	CPg-ID122	19/02/2014	2	NA	NA	NEG	2.31021°N 10.24563°W
CP9854	CPg-ID047	19/02/2014	6	NEG	980	POS	2.31021°N 10.24563°W

*ND* not done, *NA* not applicable, *POS* positive, *NEG* negative

^a^Samples individualized (ID) by microsatellite analysis [2, 20, 21]

^b^SIV-specific RT-PCR using primers CPZ-gp41 (~ 450 bp), polmini (280 bp) and gp120 V1–V4 (~ 1000 bp), as previously described [1, 21, 22, 24]

^c^Viral Load (VL), as previously described [23]

^d^Samples of the same individual

### Library preparation, quantification and NGS

The NucliSens Magnetic Extraction kit (BioMerieux, Craponne, France) and QIAamp Stool DNA Miniprep Kit (QIAGEN, Courtaboeuf, France) were used to extract nucleic acids (total nucleic acids or DNA, respectively) as previously described [20, 21]. Total nucleic acids were initially treated with the TURBO DNA-free™ kit (Life Technologies, CA, USA) and purified using the RNeasy^®^ MinElute^®^ Cleanup kit (QIAGEN), following the manufacturers’ instructions. For removal of ribosomal RNA (from plant, bacteria and mammalian host) prior to RNA library preparation, total purified RNA was subjected to the ScriptSeq™ Complete Gold Kit: Epidemiology (Epicentre, Wisconsin, USA). The MinElute PCR Purification Kit (QIAGEN) and the Agencourt AMPure XP System: PCR Purification kit (Beckman Coulter, CA, USA) were used to purify PCR-amplified products whenever requested by the ScriptSeq™ kit protocol. DNA libraries were constructed using the Nextera^®^ DNA Sample Preparation kit (Illumina^®^, CA, USA), following the manufacturer’s instructions. For all RNA and DNA library preparations, an average of 2 μg and of 50 ng of purified nucleic acid were used for each sample library, respectively. Quality control and absolute quantification of libraries were carried out in the ECO™ system (Eco Real-Time PCR System, Illumina^®^) using the KAPA Library Quantification kit (KAPA Biosystems, MA, USA), according to the manufacturers’ specifications. Libraries were diluted to a final concentration of 12 pM and NGS by synthesis was carried out in an Illumina^®^ HiSeq 2500 platform (2 × 100 paired-end runs). Sequencing data files are available in the SRA database under *BioProject* accession PRJNA419744 (BioSample accesion: SAMN08143625—SAMN0814362547).

### Bioinformatics and statistical analyses

Quality analysis of the generated sequencing reads was conducted with FastQC (Babraham Bioinformatics, Cambridge, UK) and reads with good quality (Phred score > 30 and ≥ 90 bp) were selected with Sickle-Master [25]. Reads were submitted to a BLASTX search [26] onto the GenBank nonredundant viral protein database, with a minimal e-value cutoff of 1e^−5^, and reads assigned to viruses were kept for further analysis. Virus taxonomic assignment was carried out using the Lowest-Common Ancestor (LCA) algorithm in MEGAN v. 6.3.5, built 4 (April 2016). The following LCA parameters were used: Min Score 50; Max Expected 1 × 10^−5^; Top Percent 10; Min Support Percent 75; Min Support 5; Min Complexity 0.3. Sequences without any significant hit were designated as unassigned. The MEGAN’s square-root normalization protocol enabled intra- and intergroup comparisons. We compared the virus taxa distribution between SIVgor-infected (POS) and uninfected (NEG) animals. For this and onward analyses, annotated reads of the two samples from the same SIVgor-infected animal (CPg-ID074) were pooled and averaged. Assigned sequence counts per taxa were exported for subsequent statistical analysis using GraphPad Prism v.7. Fisher’s exact test with Bonferroni correction was used for multiple comparisons. Data were presented as heatmaps with outlier data points removed whenever specified. All graphs were generated using Microsoft Excel^®^ for Mac and R Studio v. 0.99.902. Results were considered statistically significant when p values were < 0.01.

## Results

### Viral diversity in enteric samples of western lowland gorillas

**Fig. 1:**
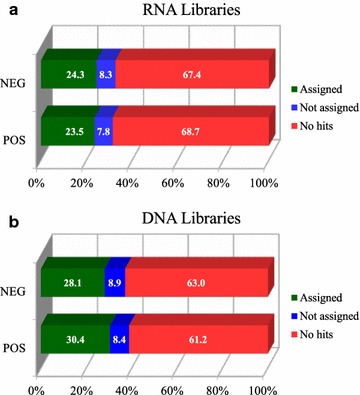
Viral taxon assignment. Percentage distribution of total reads with RNA (**a**) and DNA (**b**) libraries for SIVgor-infected (POS) and uninfected (NEG) individuals

To assess the diversity of the enteric virome of wild gorillas, RNA and DNA libraries of 23 fecal samples from 11 SIVgor-infected and 11 uninfected western lowland gorillas were sequenced. We recovered 473,357,985 high-quality reads (mean = 20,580,781.96/sample, ranging from 4386,718 to 94,513,993) for all RNA libraries and 559,920,656 reads (mean = 24,344,376.35/sample, ranging from 2511,994 to 125,855,508) for all DNA libraries. After applying MEGAN square-root normalization, a reduction in read counts to 132,138.5 reads (mean = 6006.3/sample, ranging from 2575 to 14,064) was achieved for RNA libraries and to 150,539 reads (mean = 6842.7/sample, ranging from 2409 to 23,131) for DNA libraries. BLASTX analysis showed that approximately 24 and 29% of normalized reads could be annotated for identifying virus families using a viral database for RNA and DNA libraries, respectively (Fig. 1).

The Siphoviridae bacteriophage family was more frequent in SIVgor-infected individuals (32.7 vs 23.4% in uninfected individuals), while two other bacteriophage families were more frequent in uninfected individuals, Myoviridae (31.7 vs 25.8% in infected individuals) and Podoviridae (12.9 vs 9.0% in infected individuals). Altogether, these three viral families accounted for 67.5 and 68% of the total annotated reads in SIVgor-infected and uninfected individuals, respectively (Table 2). The majority of the other viral families showed frequencies below 1% of total reads. Viral family profiles diverged significantly between the two samples collected from a single individual (CPg-ID074; data not shown), a finding that precluded further analysis.

**Table 2:** Total number of annotated reads of viral families in SIVgor-infected (POS) and uninfected (NEG) individuals

Family	POS (%)	NEG (%)
Siphoviridae	12,433.5 (32.66)	8818 (23.39)
Myoviridae	9821 (25.80)	11,939 (31.66)
Podoviridae	3439 (9.03)	4859 (12.89)
Mimiviridae	2464.5 (6.47)	2536 (6.73)
Phycodnaviridae	2452.5 (6.44)	2455 (6.51)
Unclassified dsDNA phages	1266 (3.33)	1405 (3.73)
Unclassified phages	2477.5 (6.51)	2146 (5.69)
Unclassified dsDNA viruses	821 (2.16)	847 (2.25)
Unclassified Caudovirales	491.5 (1.29)	487 (1.29)
Herpesviridae	401.5 (1.06)	356 (0.94)
Others^a^	2006 (5.27)	1857 (4.93)
Total	38,074 (100)	37,705 (100)

^a^Other viral families with abundance frequency below 1% of annotated reads: Adenoviridae; Alloherpesviridae; Alphaflexiviridae; Ascoviridae; Asfarviridae; Baculoviridae; Betaflexiviridae; Bicaudaviridae; Bromoviridae; Caulimoviridae; Circoviridae; Closteroviridae; Dicistroviridae; Hytrosaviridae; Inoviridae; Iridoviridae; Leviviridae; Marseilleviridae; Mesoniviridae; Microviridae; Nudiviridae; Parvoviridae; Picobirnaviridae; Polydnaviridae; Potyviridae; Poxviridae; Reoviridae; Retroviridae; Rhabdoviridae; Rudiviridae; Streptococcaceae Virus; Tectiviridae; Tymoviridae; Unclassified ssDNA Viruses; Unclassified ssRNA Negative-strand Viruses; Unclassified ssRNA Positive-strand Viruses; Unclassified Viruses; and Virgaviridae

### Association of gorilla virome profiles with SIVgor status

**Fig. 2:**
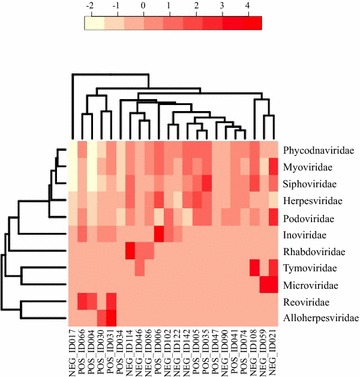
Unsupervised clustering analysis of viral diversity profiles of SIVgor-infected (POS) and uninfected (NEG) groups, showing proxies for SIVgor infection like Reoviridae and Alloherpesviridae, associated with the POS group. Tymoviridae, Microviridae and Rhabdoviridae were associated with the NEG group

We compared the virome of the SIVgor-infected and uninfected gorillas and investigated the potential association of SIV status with virome profiles. As some viral families were represented in only one or two individuals of each group, outlier data were excluded from analysis to avoid spurious associations. In the SIVgor-infected group, 22 viral families were identified, with three taxa (Alloherpesviridae, Polydnaviridae and Reoviridae) exclusively represented in this group. Conversely, 21 viral families were identified among the uninfected gorillas, with two exclusive taxa (Microviridae and Tymoviridae). Despite the restrictive criteria of excluding outliers, we found a distinct profile for the SIVgor-infected group, with two significantly more abundant mammalian viral families (Herpesviridae and Reoviridae; p < 0.003; Table 3). The uninfected group also showed a distinct virome profile, with one significantly more abundant mammalian viral family (Rhabdoviridae; p < 0.003; Table 3). Unsupervised clustering analysis with the virome profiles of all 22 individuals revealed that some viral families served as putative proxies for SIVgor infection status, like Reoviridae and Alloherpesviridae in the SIVgor-infected group, and Tymoviridae, Microviridae and Rhabdoviridae in the uninfected group (Fig. 2).

**Table 3:** Comparison of viral family abundance between SIVgor-infected (POS) and uninfected (NEG) individuals after outlier exclusion

Virus Family	POS (%)	NEG (%)	p value^a^
Alloherpesviridae	15 (0.07)	0 (0.00)	*0.0024*
Baculoviridae	32 (0.15)	50 (0.20)	0.9976
Herpesviridae	257 (1.19)	198 (0.79)	*0.0024*
Inoviridae	45 (0.21)	18 (0.07)	*0.0024*
Iridoviridae	39 (0.18)	15 (0.06)	0.2105
Marseilleviridae	53 (0.25)	66 (0.26)	1
Microviridae	0 (0.00)	22 (0.09)	*0.0024*
Mimiviridae	1838 (8.52)	2036 (8.15)	0.981
Myoviridae	6577 (30.47)	8645 (34.62)	*0.0024*
Nudiviridae	108 (0.50)	112 (0.45)	1
Parvoviridae	76 (0.35)	96 (0.38)	1
Phycodnaviridae	1759 (8.15)	1811 (7.25)	*0.0072*
Picobirnaviridae	35 (0.16)	64 (0.26)	0.5608
Podoviridae	1725 (7.99)	2322 (9.30)	*0.0024*
Polydnaviridae	2 (0.01)	0 (0.00)	0.9969
Poxviridae	19 (0.09)	44 (0.18)	0.235
Reoviridae	23 (0.11)	0 (0.00)	*0.0024*
Retroviridae	251 (1.16)	262 (1.05)	0.9989
Rhabdoviridae	2 (0.01)	32 (0.13)	*0.0024*
Siphoviridae	5666 (26.25)	5764 (23.08)	*0.0024*
Tymoviridae	0 (0.00)	65 (0.26)	*0.0024*
Unclassified Caudovirales	264 (1.22)	261 (1.05)	0.8284
Unclassified dsDNA Phages	831 (3.85)	1005 (4.02)	1
Unclassified dsDNA Viruses	575 (2.66)	603 (2.41)	0.8993
Unclassified Phages	1389 (6.44)	1479 (5.92)	*0.0024*
Total	21,582 (100)	24,970 (100)	

^a^p values were estimated with Fisher’s Exact Test with Bonferroni correction (significant values are shown in italic)

### Detection of disease-associated viruses in SIVgor-infected gorillas

A specific assessment of mammalian viral families previously associated with intestinal disease in their hosts was conducted in the studied specimens. Adenoviridae reads were found only in two SIVgor-infected gorillas. The association of this specific family with prolonged infections was characterized in CPg-ID004 (VL of 655 cp/mL) and CPg-ID047 (VL of 980 cp/mL) individuals, known to be SIVgor-infected since 2007 and 2011, respectively [20, 21]. Mammalian viral families that have been associated with gut and other diseases in humans [27–44] were also significantly more abundant in the SIVgor-infected group (p < 0.003), like Herpesviridae and Reoviridae, while only the Rhabdoviridae was more abundantly recovered in the uninfected group (p < 0.003).

## Discussion

Studies on natural history of lentiviral infections in apes have been mostly limited to captive or semi-free ape communities due to the restrictions of working in the wild and to the endangered status of these species. However, noninvasive techniques developed during the last 30 years allowed for the study of prevalence, diversity and impact of SIV infection in several wild primate populations [6]. These advances prompted us to assess novel dysbiosis markers in previously studied wild gorilla communities, using fecal samples collected in CP.

In this study, 24 and 29% of normalized reads from RNA and DNA libraries, respectively, were annotated using a virus database. These rates were in agreement with similar reports on the complexity of virome annotation [31] and the finding of a large proportion of reads frequently found in the “No Hit” category in fecal samples [28]. These drawbacks may persist even when pretreatment for reducing bacterial or general eukaryotic material is carried out, because a substantial amount of non-viral sequences can still be found in fecal samples. It is also important to consider that a substantial proportion of the fecal virome is related to dietary components and associated viruses, like plant viruses [19, 45, 46]. In our study, a large number of annotated reads was related to non-mammalian viruses, mostly bacteriophages and plant viruses. Currently, the convergence of bacterial communities of sympatric individuals living off the same diet has been well established [19, 45, 47]. Moreover, the bacteriophage community, intrinsically linked with the bacteriome, can be an indicator of the general stability of the gut microbiome in a disease context. Mammalian viruses, on the other hand, might be important markers of disease progression despite their low frequency found during annotation.

In this study, we analyzed for the first time the enteric virome of gorillas in association with SIVgor status. This also provided the first evidence of association of specific mammalian viral families with SIVgor infection in a putative dysbiosis context. This was the case of SIV-infected rhesus macaques that presented unsuspected adenovirus infection associated with intestinal disease and enteric epithelial pathology, as well as enteric parvoviruses and advanced AIDS [19]. In the gorillas herein studied, adenoviruses were only recovered from SIVgor-infected individuals, while parvoviruses were differently represented in samples with higher VL (≥ 1000 copies/mL) than with lower VL (< 1000 copies/mL) (data not shown), suggesting that parvovirus infection might vary according to VL. The picobirnaviruses recovered in this study also showed to be more frequent in animals with higher VL than with lower VL, while reoviruses were significantly more abundant in SIVgor-infected animals than in their uninfected counterparts. Infection by picobirnaviruses and reoviruses has been associated with gastrointestinal complications in advanced HIV infections [29, 30, 41, 43]. Herpesviruses were also significantly more abundant in SIVgor-infected gorillas; these viruses have already been described in AIDS patients [36–40, 42] indicating persistent infections. These findings strongly suggest that SIVgor infection might be associated with dysbiosis, although further studies will be required for validation.

Epidemiological surveys showed that SIVgor as well as SIVcpz are less prevalent and considerably less evenly distributed among wild ape communities than the non-pathogenic SIVs in sooty mangabeys and African green monkeys [6]. These distribution disparities raise the possibility that apes may not be the natural reservoirs of these viruses, and that might be subjected to pathogenic conditions similar to those in other non-natural lentiviral hosts like humans and rhesus macaques [15, 19, 48, 49]. Currently SIVcpz, like HIV-1, is known to cause significant morbidity and mortality in infected chimpanzee communities [12, 13, 50], in association to compositional changes of the bacteriome rather than the virome, with disease progression with known (or suspected) immunodeficiency in late stages [51]. A similar study in wild gorillas failed to show association between SIVgor status and the bacteriome [52], while data on their virome has been, to present, wanting.

This study is underpinned by important limitations. In the great apes, it is well known that the gut microbiome community and abundance profiles are shaped by individual and external factors, like sex, age, time elapsed since fecal deposition, and dietary habits related with the rain or dry seasons of CP. With respect to sex, samples of SIVgor-infected and uninfected females showed larger numbers of reads of families of bacterial, amoebal, algal, invertebrate, and vertebrate viruses, both in SIVgor-infected and uninfected groups (not shown). In view of the small number of individuals within each group, this sex bias was not considered in further analyses. Moreover, our samples did not allow us to identify any age-related virome variation, since age estimation through noninvasive material is a very difficult, maybe unfeasible task.

Campo Ma’an is located in the southwest coast of Cameroon, a region with coastal equatorial climate where a heavy rain season (from August to November) is followed by a long dry season (from December to March), a short rain season (from April to May) and a short dry season (June and July). These alternate seasons might impose drastic diet changes resulting from different feeding strategies which, in turn, might be reflected in the fecal virome. Because we did not use seasonality as a factor for sample selection, both SIV-infected and uninfected groups had samples from dry and wet seasons, and such analysis was not considered in the present study, but deserves further assessment.

Despite the restrictive criteria herein adopted to minimize viral misidentification, the small number of studied individuals may produce imprecise estimates of microbiome profiles. Finally, the computational pipeline developed by us was characterized by a simple sequence of algorithms for identifying viral families without further characterization of full-length viral sequences or discovering novel viruses. On the other hand, the stringency for removing unreliable reads, despite hindering virus discovery, allowed robust description of the virome composition of SIVgor-infected and uninfected gorillas. However, higher number of samples will be necessary for reducing intrinsic sampling variation.

## Conclusions

We herein describe, for the first time, the virome of wild gorillas, although these preliminary observations were carried out in a small number of individuals. This study suggests that the virome of SIVgor-infected individuals might differ from the one present in uninfected animals, in contrast to what has been reported for the bacteriome in this species [18]. The microbiome of SIVgor-infected animals must be further explored to validate present findings and to confirm whether virome dysbiosis might be consistent with the characteristic disease profile observed in SIVcpz-infected chimpanzees [17], the more classic pathogenic SIVmac infection in rhesus macaques, and HIV-1 infection in humans. The diverse mammalian viral families, herein described for the first time in SIVgor-infected gorillas, may play a pivotal role in a disease progression that still remains unclear in these animals but is already well characterized in pathogenic lentiviral infections. Our results suggest that viromes might be considered as markers of lentiviral disease progression in wild gorilla populations and should be further explored on a larger sample set.
